# Classical Reaction Barriers in DFT: An Adiabatic-Connection Perspective

**DOI:** 10.1021/acs.jctc.4c01038

**Published:** 2024-12-23

**Authors:** Andrew M. Wibowo-Teale, Bang C. Huynh, Trygve Helgaker, David J. Tozer

**Affiliations:** † School of Chemistry, University of Nottingham, University Park, Nottingham NG7 2RD, U.K.; ‡ Hylleraas Centre for Quantum Molecular Sciences, Department of Chemistry, University of Oslo, P.O. Box 1033 Blindern, N-0315 Oslo, Norway; § Department of Chemistry, Durham University, South Road, Durham DH1 3LE, U.K.

## Abstract

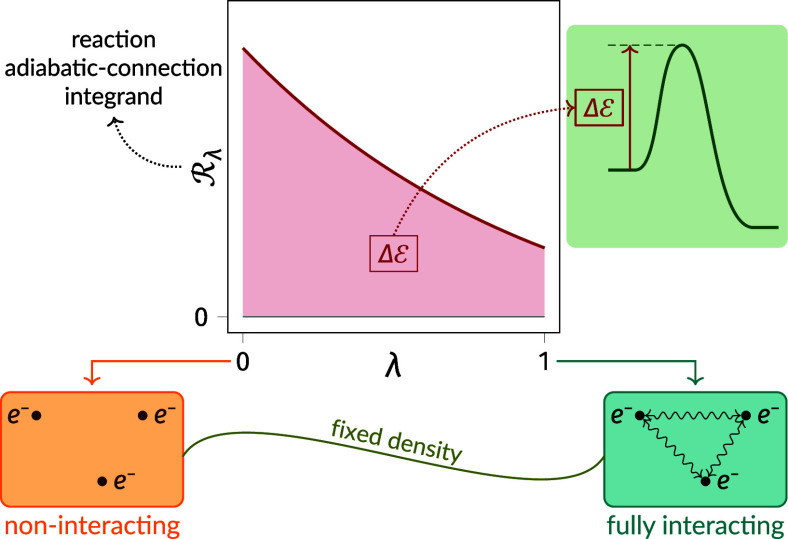

Classical reaction barriers in density-functional theory are considered from the perspective of the density-fixed adiabatic connection. A ‘reaction adiabatic-connection integrand’, , is introduced, where λ is the electron–electron interaction strength, for which  equals the barrier, meaning the barrier can be easily visualized as the area under a plot of  vs λ. For five chemical reactions, plots of reference , calculated from Lieb maximizations at the coupled-cluster level of theory, are compared with approximate , calculated from common exchange–correlation functionals using coordinate scaling, for coupled-cluster densities. The comparison provides a simple way to visualize and understand functional-driven errors and trends in barriers from approximate functionals, while allowing a clean separation of the role of exchange and correlation contributions to the barrier. Specifically, the accuracy of  is determined entirely by the accuracy of the exchange functional, while the shape of  is determined entirely by the correlation functional. The results clearly illustrate why the optimal amount of exact (orbital) exchange in hybrid functionals differs between reactions, including forward and reverse directions in the same reaction, and hence why simply introducing larger amounts of exact exchange may not be a reliable approach for improving barriers. Instead, the shape of  must be captured more accurately through more accurate correlation functionals, and the numerical data presented may be useful for this purpose. Density-driven errors are then considered, and possible cancellation with functional-driven errors in barriers—noted in prior studies when Hartree–Fock densities are used—is illustrated from the perspective of .

## 1 Introduction

Kohn–Sham density-functional theory^1^ (DFT) is the most widely used electronic-structure method, achieving remarkable accuracy—considering its relatively low cost—for a broad range of properties and quantities.^2−4^ The accuracy of a practical DFT calculation is governed by the accuracy of the exchange–correlation energy functional, *E*_xc_[ρ], where ρ is the electron density. Numerous density functional-approximations (DFAs) to this quantity have been proposed.

One chemical quantity that remains a particular challenge for DFAs, especially semilocal approximations, is the classical reaction barrier,
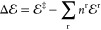
1where  is the total energy (electronic and nuclear repulsion) of the transition state (‡) and the summation is over reactants (r) with total energies, , weighted by their stoichiometries, *n*^r^. We shall, hereafter, simply refer to it as a “barrier”. It is well established that DFAs often significantly underestimate barriers and this deficiency has been analyzed in terms of the one-electron self-interaction error^5−18^ and many-electron self-interaction or delocalization errors.^19−23^ A common approach for improving the accuracy of barriers from DFAs is to increase the amount of exact (orbital) exchange,^13,24^ to yield hybrid DFAs. Alternatively, the use of explicit self-interaction corrections has been explored.^10,11,16−18,25,26^

While the perspectives of self-interaction and delocalization errors give important insight into how errors in barriers arise, they do not distinguish clearly between the role of errors in the exchange and correlation components of *E*_xc_[ρ]. The aim of the present work is to consider barriers in DFT from a new perspective, namely that of the density-fixed adiabatic connection,^27−30^ which does clearly distinguish the effects of exchange and correlation. This approach, which connects the Kohn–Sham noninteracting electronic system (λ = 0) with the physical interacting system (λ = 1) through a series of partially interacting systems with electron–electron interaction strength, λ, with the same density, underpins many important aspects of DFT, including hybrid functionals^31^ and the perturbation theory of Görling and Levy.^32^

We commence in Section 2 by presenting the necessary theory and computational details. Starting from the Levy constrained-search variation principle,^33^ we define the exchange–correlation integrand, , the accurate calculation of which requires an accurate wave function for interaction strength λ. We then show how maximization of the Lieb functional^34^ using a correlated electronic-structure method can yield such a wave function and hence an accurate reference . We also describe how  can be determined for an arbitrary DFA by coordinate scaling.^35−38^ We then describe how we compute Kohn–Sham barriers, and how they relate to , and we distinguish between functional- and density-driven errors.^39^

In Section 3, we present plots of , as a function of λ, for the reactants and transition state in a simple reaction, comparing accurate reference plots, determined from Lieb maximizations using coupled-cluster singles-doubles-perturbative-triples (CCSD(T)) wave functions,^40^ with plots for common DFAs, determined using coordinate scaling for relaxed (Lagrangian) CCSD(T) densities. Consideration of the associated barriers leads us to define a ‘reaction adiabatic-connection integrand’, , involving the difference between  of the transition state and reactants, whose integral between λ = 0 and λ = 1 equals the barrier.

In Section 4, plots of reference CCSD(T) , as a function of λ, are compared with those from approximate DFAs for five chemical reactions, providing insight into the dependence on DFA, the role of exchange and correlation contributions, the effect of adding an amount of exact exchange to yield hybrid functionals, the differences between forward and reverse reactions, and the distinction between functional- and density-driven errors. Finally, some conclusions are presented in Section 5.

## 2 Theory and Computational Details

### 2.1 Levy Constrained-Search Functional

Consider an *N*-electron system with the Hamiltonian,
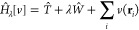
2where *T̂* is the kinetic-energy operator, *Ŵ* is the electron–electron repulsion operator, and *v* is the external potential. The ground-state electronic energy at a given interaction strength, λ ∈ [0, 1], is given by the Rayleigh–Ritz variation principle as
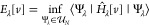
3where  is the set of all *L*^2^-normalized, antisymmetric *N*-electron wave functions with a finite kinetic energy. The ground-state energy in eq 3 is well-defined for all potentials *v* ∈ χ* with χ* = *L*^3/2^ + *L*^*∞*^, a vector space that includes all Coulomb potentials. A minimizing wave function may or may not exist in eq 3, depending on whether the external potential *v* supports an *N*-electron ground state.

Following Levy,^33^ we may express the ground-state energy of eq 3 in the form
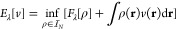
4where  is the set of all *N*-representable densities and
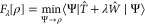
5is the constrained-search expression for the universal density functional. For each interaction strength λ and each , one or more minimizing wave functions exist in the constrained search and we may therefore write the universal density functional as an expectation value
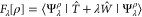
6where Ψ_λ_^ρ^ is one of the minimizing wave functions of eq 5.

### 2.2 Kohn–Sham Decomposition and Adiabatic Connection

Differentiating eq 6 with respect to λ and applying the Hellmann–Feynman theorem, we arrive at the adiabatic-connection expression for the universal density functional,^27−30^
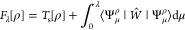
7where *T*_s_[ρ] = *F*_0_[ρ] is the noninteracting kinetic energy functional. Introducing the classical Coulomb (Hartree) energy functional, *E*_J_[ρ], and the exchange–correlation integrand
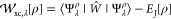
8we obtain from eq 7 the Kohn–Sham decomposition of the universal density functional,^1^
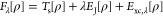
9where the exchange–correlation energy at interaction strength λ is given by
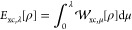
10Evaluation of *E*_xc,λ_[ρ] at λ = 1 yields the familiar exchange–correlation energy of the interacting system, *E*_xc_[ρ]. The exchange–correlation energy at interaction strength λ may be further decomposed into exchange and correlation energies
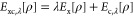
11where the exchange energy, *E*_x_[ρ], is obtained by evaluating eq 8 at λ = 0, that is, using the noninteracting wave function,
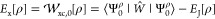
12and the remainder, *E*_c,λ_[ρ] = *E*_xc,λ_[ρ] – λ *E*_x_[ρ], is the correlation energy at interaction strength λ,
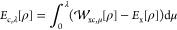
13Thus, while the exchange energy represents the (usually dominant) linear dependence of the exchange–correlation energy on the interaction strength, all (usually small) nonlinear dependence is contained in the correlation energy.

The exchange–correlation integrand,  in eq 8, is the central adiabatic-connection quantity in the present work. Our first task is to evaluate this quantity accurately, which requires us to compute Ψ_λ_^ρ^ using high-precision quantum chemistry. This, in turn, requires a consideration of the Lieb functional and its maximization.^34^

### 2.3 Lieb Functional

The (constrained-search) universal density functional in eq 5 is not directly amenable to calculation. There exists, however, an alternative formulation of the universal density functional, which is well suited to calculation and which yields the minimizing wave function Ψ_λ_^ρ^, thereby enabling the calculation of the desired  in eq 8.

Noting that the ground-state electronic energy *E*_λ_[*v*] is continuous and concave in the external potential, Lieb concluded that there exists a (convex) universal density functional *F̃*_λ_[ρ] such that^34^
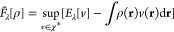
14
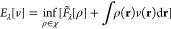
15where *F̃*_λ_[ρ] ≤ *F*_λ_[ρ] for each , with equality for all pure-state *v*-representable densities.^34^ We note in passing that, if the constrained search^33^ in eq 5 is extended to include also a search over mixed states, then full equality with the Lieb functional is obtained.^34^ Note also that, if *E*_λ_[*v*] of eq 3 is substituted in eq 14, then the external potential of the Lieb functional plays the role of a Lagrange multiplier in an unconstrained minimax optimization of the universal density functional.

For a *v*-representable density ρ, the supremum in the Lieb functional of eq 14 can be attained and there exists a potential *v*_λ_^ρ^ such that
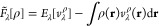
16The optimization to yield the maximizing potential *v*_λ_^ρ^ can be carried out practically^41−44^ by choosing a correlated electronic-structure method to calculate *E*_λ_[*v*] in eq 14 and setting the density ρ in eq 14 equal to the interacting density from that electronic-structure method, for all λ ∈ [0, 1]. Once the maximizing potential is obtained, the wave function Ψ_λ_^ρ^, required for the evaluation of  in eq 8, can be obtained directly from the calculation of *E*_λ_[*v*].

### Reference  from Lieb Maximizations

2.4

The potential in eq 16 depends explicitly on λ and can be decomposed in the manner used in Kohn–Sham theory,
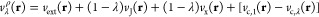
17where *v*_ext_(**r**) is the external potential due to the nuclei, *v*_J_(**r**) is the Coulomb potential from density ρ, *v*_x_(**r**) is the exchange potential, and the final term corresponds to the difference between the full correlation potential *v*_c,1_(**r**) and that at the interaction strength under consideration, *v*_c,λ_(**r**).

To facilitate the optimization in eq 14 with respect to the potential for a given density ρ, the potential is expanded in a Gaussian basis as proposed by Wu and Yang,^42,45^
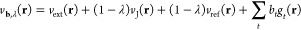
18in which *v*_ref_(**r**) is a reference potential evaluated on ρ, which ensures that *v*_**b**,λ_(**r**) has the correct asymptotic behavior, while *g*_*t*_ are a set of Gaussian functions with expansion coefficients *b*_*t*_ to be determined. The form of the reference potential employed in this work is that of the localized Hartree–Fock potential,^46^ corrected at long-range by an approximate Fukui potential.^47^ The details of the construction of the reference potential are given in ref (48).

With the parametrization of the potential in eq 18, the universal density functional in eq 14 can be calculated by maximizing the objective function,
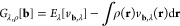
19with respect to variations in the potential-basis coefficients **b**. The gradient of eq 19 with respect to these coefficients is given by^42^
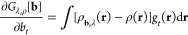
20while the second derivative of the objective function with respect to the potential-basis coefficients is given by^42^
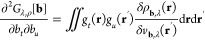
21At the maximizing potential *v*_λ_^ρ^, the iterating density ρ_**b**,λ_ becomes identical to the input density ρ.

In this work, the objective function is maximized by an approximate Newton approach^49^ combined with GDIIS^50^ as implemented in the Quest program.^51^ This is a second-order optimization algorithm in which the Hessian is approximated by the noninteracting Hessian, given by eq 21 at λ = 0.^49^ Initially, the basis coefficients of the potential are updated at each iteration using a backtracking line search and *E*_λ_ is evaluated with the corresponding potential *v*_**b**,λ_, yielding the energy and iterating density ρ_**b**,λ_ from which the objective function eq 19, gradient eq 20, and approximate Hessian are constructed. When the Euclidean norm of the gradient in eq 20 falls below 10^–4^ a.u., the GDIIS algorithm is used to achieve convergence to a final gradient norm of 10^–8^ a.u. The use of GDIIS acceleration is discussed in Appendix A and ensures that very tight convergence can be achieved at all interaction strengths. At each step, the approximate Hessian is regularized using the smoothing norm procedure of ref (52), with a regularisation parameter of 10^–5^ a.u.

All Lieb maximizations in this work are carried out at the CCSD(T)^40^ level of theory in the cc-pCVTZ basis^53−56^ for λ ∈ [0, 1]. See the Supporting Information for further details. The same basis set is used for all calculations throughout this work. The relaxed (Lagrangian) CCSD(T) density^57−59^ and the wave function Ψ_λ_^ρ^ from the λ-interacting CCSD(T) calculation in the Lieb maximization are used to calculate  in eq 8. We shall use this as our reference  throughout this work.

###  from Exchange–Correlation Energy Functionals

2.5

For any exchange–correlation functional *E*_xc_[ρ]—exact or approximate—the corresponding integrand  can be determined from coordinate scaling,^35−38^
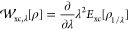
22where ρ_1/λ_ is the coordinate-scaled density,
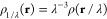
23Partitioning *E*_xc_[ρ] into exchange and correlation functionals, and using the fact that the exchange functional satisfies the coordinate-scaling condition,^35^
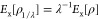
24we obtain
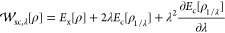
25The exchange contribution to  is therefore constant in λ and equal to the exchange energy, in agreement with eqs 10–12. Any deviation from constancy in  reflects the effect of electron correlation and arises from second- and higher-order dependence of the correlation energy on λ.

We use eq 25 to determine  for DFA energy functionals. The coordinate-scaled correlation functional *E*_c_[ρ_1/λ_] can be readily evaluated for a given DFA using the scheme described in ref (60). In this approach, the ingredients defining the functional are coordinate scaled to evaluate *E*_c_[ρ_1/λ_]; expressions for these quantities may be found in eqs 49–53 of ref (60). Some care is required to ensure accurate numerical integration of the scaled quantities as λ approaches zero. In practice, we use a relatively fine numerical quadrature grid consisting of the order-41 Lebedev angular grid and a radial component constructed using the scheme of Lindh, Malmqvist, and Gagliardi^61^ with a relative error threshold of 10^–10^ a.u. Once *E*_c_[ρ_1/λ_] has been determined, the λ-derivative in the final term of eq 25 is evaluated by a simple forward finite difference with a step size of 10^–6^ in λ.

This procedure has been implemented in the Quest program^51^ and is available for all DFAs in the XCFun^62^ or LibXC^63^ libraries. For a given DFA, the implementation allows eq 25 to be evaluated for any available density; we consider the self-consistent Kohn–Sham density of the DFA under study, the Hartree–Fock density, and the reference CCSD(T) density. For meta-GGA functionals and functionals containing exact (orbital) exchange, the evaluation of eq 25 requires a knowledge of the orbitals corresponding to the density. For the Kohn–Sham and Hartree–Fock densities, we use the orbitals from standard self-consistent field calculations. For the reference CCSD(T) density, the orbitals are obtained from a CCSD(T) Lieb maximization, as described in Section 2.4, at λ = 0. The optimizing potential of eq 17 is, in this case, the Kohn–Sham effective potential *v*_s_, yielding Kohn–Sham orbitals and orbital energies consistent with the CCSD(T) density.

### 2.6 Kohn–Sham Barriers

Next, consider Kohn–Sham barriers and how they relate to . In the Kohn–Sham decomposition,^1^ the total energy at interaction strength λ takes the form
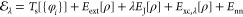
26where *T*_s_[{φ_*i*_}] is the noninteracting kinetic energy calculated from the occupied Kohn–Sham orbitals φ_*i*_,  is the interaction energy between the density and the external potential, and *E*_nn_ is the nuclear repulsion energy, which depends only on the nuclear coordinates and charges. Inserting eq 26 into eq 1 and using eq 10, the barrier at interaction strength λ can be written as
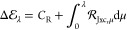
27where the first term contains the Kohn–Sham contributions to the barrier that are independent of the interaction strength,
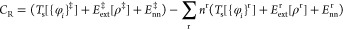
28while the second term contains the exchange–correlation and Coulomb contributions to the barrier, calculated by interaction-strength integration over the integrand,
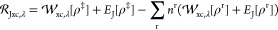
29Note that the use of two superscripts in *E*_ext_^‡^[ρ^‡^] and  in eq 28 emphasizes that both the density and external potential depend on the molecular structure.

For a given DFA, we use the Quest program^51^ to evaluate the barrier in eq 27 for the self-consistent Kohn–Sham density of the DFA under study, the Hartree–Fock density and the reference CCSD(T) density.

For the self-consistent Kohn–Sham density, the *T*_s_, *E*_ext_, and *E*_J_ terms in eqs 28 and 29 are calculated directly from the self-consistent density and corresponding orbitals. The  terms in eq 29 are evaluated for the self-consistent density, as described in Section 2.5, for λ ∈ [0, 1] in steps of 0.05. The integral in eq 27 is then evaluated numerically using these data. We have confirmed that, for the systems considered in this work, the physical barriers (eq 27 for λ = 1) determined in this manner agree with the conventionally calculated DFA barriers to better than 0.1 kcal mol^–1^; the high accuracy follows from the fact that  is a smooth function of λ.

For the Hartree–Fock density, exactly the same procedure is followed, except that the Hartree–Fock density and orbitals are used throughout, instead of self-consistent Kohn–Sham ones. For the reference CCSD(T) density, we again use the same procedure, but now use the CCSD(T) density and orbitals from a CCSD(T) Lieb maximization at λ = 0, throughout.

In addition to DFA barriers, we also calculate Kohn–Sham barriers purely from CCSD(T) data, which we shall use as reference barriers throughout this work. To do this, we again calculate the *T*_s_, *E*_ext_, and *E*_J_ terms in eqs 28 and 29 directly from the CCSD(T) density and orbitals from a CCSD(T) Lieb maximization at λ = 0. However, for , we use the reference CCSD(T) values of Section 2.4, numerically integrating them as above. Physical barriers determined in this manner agree with standard CCSD(T) barriers—that is, those obtained without any consideration of DFT—to within 0.2 kcal mol^–1^.

It is worth noting that the values of *C*_R_ and the *E*_J_ contribution to  are identical for DFA calculations for CCSD(T) densities and for the reference CCSD(T) calculations. This will be pertinent in Sections 3 and 4.

### 2.7 Functional-Driven and Density-Driven Errors

The flexibility to evaluate  and Kohn–Sham energy components for various densities (including high-accuracy reference densities) allows for quantification of two distinct sources of error: those arising from errors in the density and those arising from errors in the DFA. Following Kim et al.,^39^ we write the error in a given quantity, *Q*, obtained from a self-consistent calculation using a DFA, as
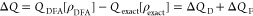
30where the *density-driven* error,
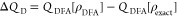
31arises from the error in the density when a DFA is used, while the *functional-driven* error,
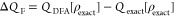
32arises from the error in the DFA when the exact density is used. In the context of barriers, we note that *C*_R_ in eq 28 can only have density-driven errors, whereas  in eq 29 may carry functional-driven, as well as density-driven errors.

The main focus of this work will be on functional-driven errors, although we shall consider density-driven errors in Section 4.2. We use the reference CCSD(T) densities as a sufficiently accurate proxy for ρ_exact_ in eqs 31 and 32.

## 3 Reaction Adiabatic-Connection Integrand

**Figure 1:**
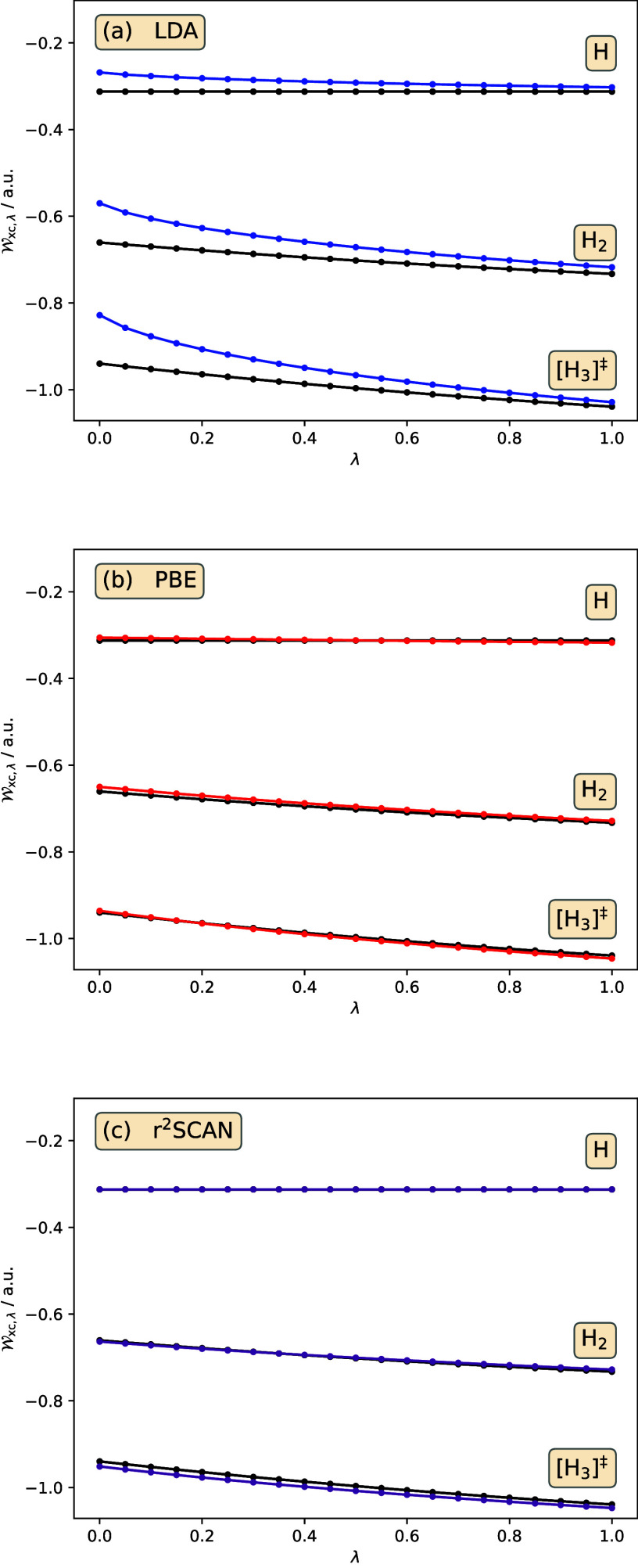
for reactants and transition state of the reaction H + H_2_ → H_2_ + H, as a function of interaction strength λ, for reference CCSD(T) (black) and (a) LDA (blue), (b) PBE (red), (c) r^2^SCAN (purple). All DFA  are calculated for CCSD(T) densities.

To illustrate the adiabatic connection in the context of a chemical reaction, Figure 1 presents the reference CCSD(T) integrand , as a function of λ, for the reactants and transition state in the exchange reaction of H with H_2_ to form H_2_ and H. These are compared with the corresponding (a) LDA,^64,65^ (b) PBE,^66^ and (c) r^2^SCAN^67^ integrands, determined by evaluating eq 25 for CCSD(T) densities. The difference between the DFA and reference CCSD(T) curves therefore quantifies the functional-driven error, eq 32, in . These DFAs were chosen as representative of the first, second, and third rungs, respectively, of Jacob’s Ladder of DFAs.^68^ The r^2^SCAN functional was selected as the representative meta-GGA because it satisfies a wide range of exact constraints while overcoming numerical instabilities associated with the original SCAN functional.^69^ (We have also reproduced all calculations in this paper with the TPSS meta-GGA functional^70^ and the results are very similar to those of r^2^SCAN.) We employ reference geometries from the BH76 set in the GMTKN55 database.^71−74^

The reference CCSD(T) curve for the H atom is horizontal, reflecting the fact that there is no electron correlation in a one-electron system and so , see eq 25. By contrast, the reference curves for H_2_ and H_3_ are not horizontal, reflecting the presence of correlation in these systems. However, neither curve exhibits a significant curvature, indicating primarily dynamic correlation, consistent with low-order dependence on the interaction strength, which can be well described by low-order Görling–Levy perturbation theory from their noninteracting Kohn–Sham systems.^32,43,44^

The LDA curves in Figure 1a are in relatively poor agreement with the reference CCSD(T) ones, with all three exhibiting excessive curvature at low λ. For the H atom, the deviation from horizontal reflects a spurious self-interaction in the LDA correlation functional. The PBE curves in Figure 1b are a notable improvement over LDA, although the curve for the H atom is still not quite horizontal, again reflecting spurious self-correlation. By contrast, the r^2^SCAN curve for the H atom in Figure 1c is horizontal and equal to the reference curve, while the curves for H_2_ and H_3_ resemble those of the PBE functional. The PBE curve is closest to the reference curve for H_3_, whereas r^2^SCAN is closest for H_2_.

In order to quantify the functional-driven errors in the associated barriers, we must compare the DFA barriers determined for CCSD(T) densities with the reference CCSD(T) barriers. From Section 2.6, these DFA and reference barriers both involve the same *C*_R_ in eq 28 and the same *E*_J_ contribution to  in eq 29. Given that the only other contribution to the barrier in eq 27 arises from  in eq 29, it follows that the differences between the DFA and reference barriers arise entirely due to the differences between the black and colored curves in Figure 1.

Given the relatively subtle differences between the curves in Figure 1, we might hope to obtain relatively accurate DFA barriers—in particular, for PBE and r^2^SCAN. However, when eq 27 is evaluated for the physical system (λ = 1), barriers of −3.0, 3.5, 2.5, and 10.0 kcal mol^–1^ are obtained for LDA, PBE, r^2^SCAN, and CCSD(T), respectively. The DFA barriers are therefore in very poor agreement with the reference CCSD(T) barrier.

This simple example illustrates that analysis of the individual  plots provides limited insight into the associated barriers. This is, of course, unsurprising given that the plots relate to relatively large individual exchange–correlation energies via eq 10, whereas the barriers relate to relatively small total-energy differences via eq 1. It is therefore instructive to instead consider the  integrand in eq 29*directly*. Given that our interest is in barriers of physical systems (λ = 1), it is convenient to introduce the function,
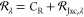
33for which
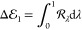
34meaning the “area” between the  curve and the horizontal axis , between λ = 0 and λ = 1, equals the barrier of the physical system, eq 27 at λ = 1. Note that replacing the upper integral limit in eq 34 by an arbitrary λ ≠ 1 would *not* yield  because *C*_R_ would be incorrectly scaled by λ. We denote  a *reaction adiabatic-connection integrand* and we investigate its utility in Section 4.

##  for Representative Reactions

4

The hydrogen transfer reaction set, HTBH38,^71^ and non-hydrogen transfer reaction set, NHTBH38,^72^ of Truhlar and co-workers have been extensively used to evaluate the performance of DFAs, often combined in the BH76 set in the GMTKN55 database.^71−74^ We consider a subset of five of these reactions, listed in Table 1, performing all calculations at the database reference geometries. For each reaction, the table presents high-accuracy benchmarks barriers at the W2-F12 level of theory for the GMTKN55 database.^74,75^ Also shown are standard CCSD(T) barriers. In all cases, the CCSD(T) barriers agree with the benchmark W2-F12 barriers to better than 1.5 kcal mol^–1^, confirming that the CCSD(T)/cc-pCVTZ level of theory is of sufficient accuracy. Preliminary calculations highlighted the need for triple excitations to achieve this level of agreement. A detailed discussion of basis-set convergence of the reaction barriers at the coupled-cluster level can be found in the Supporting Information.

**Table 1:** Benchmark W2-F12^74,75^ and CCSD(T) Forward and Reverse Barriers^a^

reaction	W2-F12		CCSD(T)	
	forward	reverse	forward	reverse
H + H_2_ → H_2_ + H	9.7	9.7	10.0	10.0
H + N_2_ ⇄ HN_2_	14.6	10.9	15.6	10.2
HCN ⇄ CNH	48.1	33.0	48.0	33.0
H_2_ + OH ⇄ H_2_O + H	5.2	21.6	6.6	20.6
H_2_ + CH_3_ ⇄ H + CH_4_	11.9	15.0	12.2	15.3

a All values in kcal mol^–1^.

### Functional-Driven Errors in  and Barriers

4.1

We commence by comparing plots of reference CCSD(T) , eq 33, as a function of λ, and their associated barriers, eq 34, with those determined from DFAs for CCSD(T) densities. All terms are calculated as described in Section 2.6. Differences again quantify functional-driven errors. Studies^76−78^ indicate that functional-driven errors in barriers are typically negative and of significant magnitude for semilocal DFAs.

#### 4.1.1 H + H_2_ → H_2_ + H

**Figure 2:**
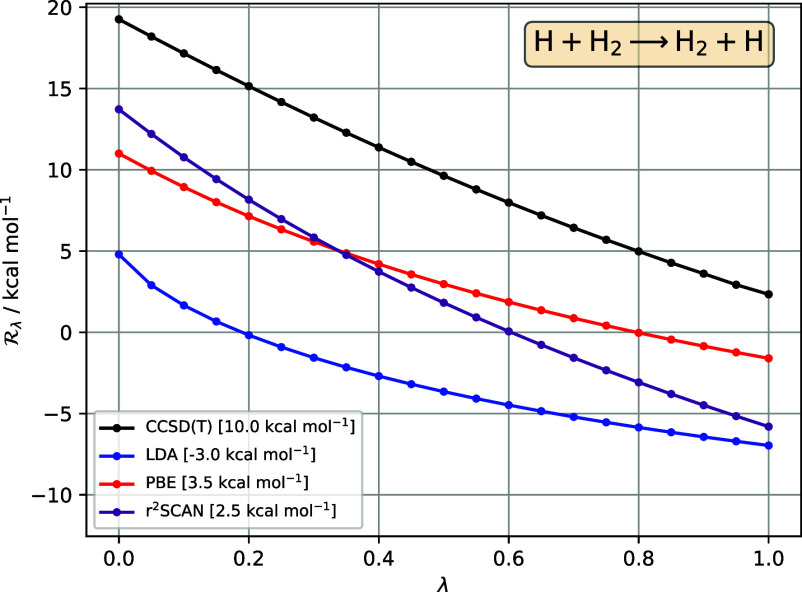
in eq 33, for the reaction H + H_2_ → H_2_ + H, as a function of interaction strength λ, for reference CCSD(T) (black), LDA (blue), PBE (red), r^2^SCAN (purple). All DFA  are calculated for CCSD(T) densities. The barrier for each method—given in the legend—is the area between the  curve and the horizontal axis .

First, we revisit the exchange reaction considered in Section 3. Figure 2 presents the reference CCSD(T) , compared to approximate  for LDA, PBE, and r^2^SCAN. Note that the vertical scale is now in kcal mol^–1^.

The area between the reference CCSD(T) curve and the horizontal axis is the aforementioned reference barrier of 10.0 kcal mol^–1^. The LDA curve is significantly below the reference curve and the *negative* area/barrier (−3.0 kcal mol^–1^) is clearly evident. The PBE and r^2^SCAN curves more closely resemble the reference curve, with correspondingly larger areas/barriers, although errors remain significant; it is clear from the areas that the PBE barrier (3.5 kcal mol^–1^) is slightly larger and more accurate than that of r^2^SCAN (2.5 kcal mol^–1^), although both still strongly underestimate the reference value. Figure 2 clearly illustrates negative functional-driven errors, consistent with refs (76−78).

As well as providing a simple way to “visualize” the barrier, a key feature of  computed in this manner is that it allows a clean separation of the role of exchange and correlation contributions to the barrier, due to the scaling property in eq 25. From eqs 25, 28, 29, and 33, together with the fact that all methods yield the same value of *C*_R_ and the same *E*_J_ contribution to , *the accuracy of* (*i.e., the left-hand points in*Figure 2) *is determined entirely by the accuracy of the exchange DFA*. The value of  in Figure 2 is strongly underestimated for LDA, somewhat improved for PBE and improved further for r^2^SCAN, although the discrepancy remains significant. By contrast, *the shape of**is determined entirely by the correlation DFA*. It is clear from Figure 2 that the behavior of LDA correlation under the scaling relation of eq 25 is poor, reflected by relatively strong curvature in  compared with the CCSD(T) reference curve. For PBE correlation,  is less curved but is still far from parallel to the reference curve. For r^2^SCAN correlation,  becomes most parallel to the reference curve.

Interestingly, despite the aforementioned improvements in both  and the shape of  from PBE to r^2^SCAN, the barrier (area) actually degrades because the increase in barrier associated with the improved exchange (improved ) is more than compensated by the decrease in barrier associated with the improved correlation (improved shape of ). Put another way, PBE benefits from error cancellation between exchange and correlation, whereas r^2^SCAN does not.

**Figure 3:**
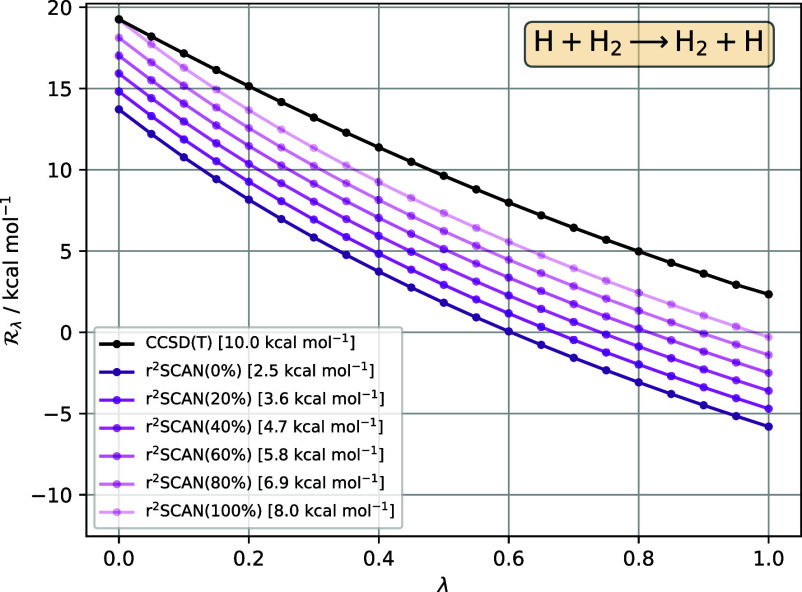
in eq 33, for the reaction H + H_2_ → H_2_ + H, as a function of interaction strength λ, for reference CCSD(T) (black) and r^2^SCAN hybrid functionals, with varying amounts of exact (orbital) exchange indicated in parentheses (purple lines, lighter shading indicates more exact exchange). The pure r^2^SCAN functional is written as r^2^SCAN(0%). All DFA  are calculated for CCSD(T) densities. The barrier for each method—given in the legend—is the area between the  curve and the horizontal axis .

It is well-known that introducing an amount of exact (orbital) exchange into an approximate functional, to yield a hybrid functional, often increases the value of, and hence improves the accuracy of barriers. The aforementioned separation of the role of exchange and correlation contributions in  makes the introduction of exact exchange particularly simple to visualize: as the amount of exact exchange increases from 0 to 100%, the shape of  is unchanged, but the curve shifts vertically, such that  shifts from that of the 0% functional to that of the reference CCSD(T) curve. This is illustrated in Figure 3, which plots  for hybrid versions of the r^2^SCAN functional, from 0% (conventional r^2^SCAN) to 100%. The optimal barrier (area) of 8 kcal mol^–1^ is obtained with 100% exchange. (We only consider amounts in the range 0% – 100% in this work). To address the remaining 2 kcal mol^–1^ error in a rigorous manner, it is necessary to consider improvements to the correlation DFA in order to fix the shape of . Access to accurate  may therefore be desirable for functional development and testing.

The reaction of H with H_2_ is a simple case, which is symmetric in the forward and reverse directions. We now consider other representative reactions from the BH76 set to illustrate the utility of  more broadly.

#### 4.1.2 H + N_2_ ⇄ HN_2_

**Figure 4:**
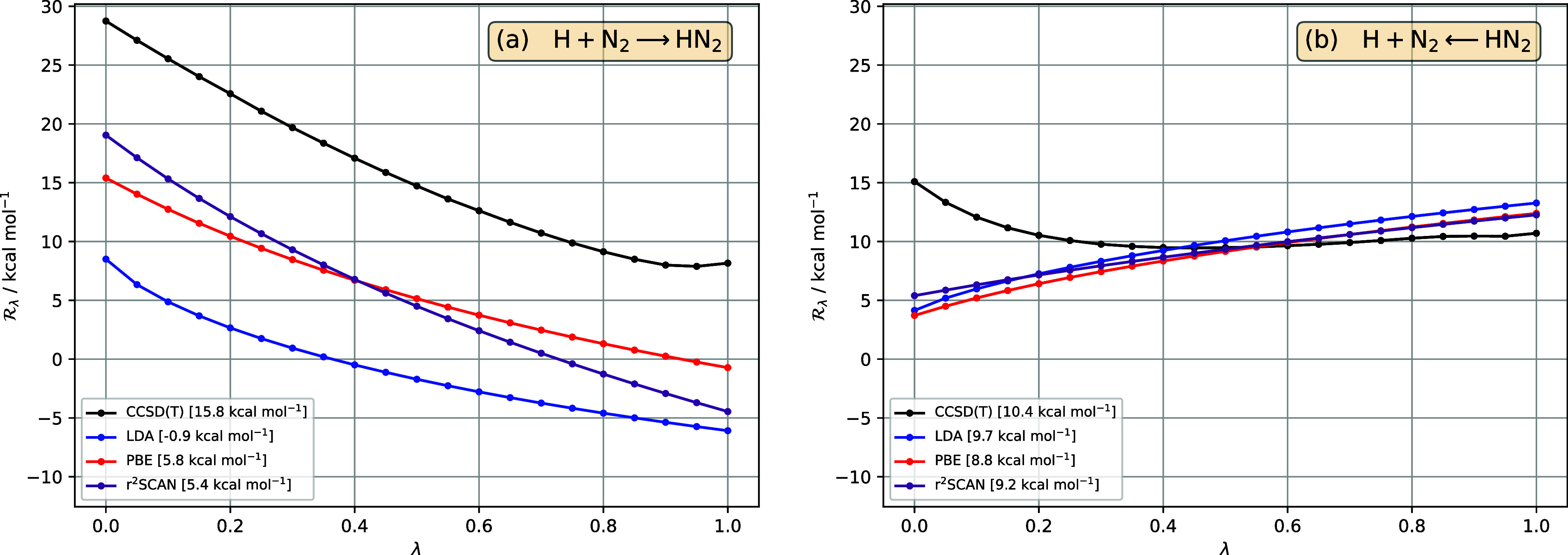
in eq 33, for the reaction H + N_2_ ⇄ HN_2_, as a function of interaction strength λ, in (a) forward and (b) reverse directions, for reference CCSD(T) (black), LDA (blue), PBE (red), r^2^SCAN (purple). All DFA  are calculated for CCSD(T) densities. The barrier for each method—given in the legend—is the area between the  curve and the horizontal axis .

As an example of an asymmetric reaction from the BH76 set, where the forward and reverse directions are different, we consider the addition reaction of H with N_2_ to form HN_2_ and the reverse dissociation process. Figure 4a,b present the reference CCSD(T)  for the forward and reverse reactions, respectively, compared to approximate  for LDA, PBE, and r^2^SCAN.

First, consider the forward reaction H + N_2_ → HN_2_ in Figure 4a. In moving from LDA to PBE to r^2^SCAN, the behavior closely resembles what was observed for H + H_2_ → H_2_ + H, with an improvement in  and the shape of the  curve from LDA to PBE to r^2^SCAN, but a slight degradation in the barrier from PBE to r^2^SCAN.

By contrast, for the reverse reaction HN_2_ → H + N_2_ in Figure 4b, all three DFAs yield rather similar  curves, none of which bear much resemblance to the reference CCSD(T) curve. The areas between these curves and the horizontal axis are, however, reasonably close to that of the reference curve due to error cancellation between low- and high-λ regions. Hence the barriers are reasonably good—LDA is actually best—with a maximum error of 0.7 kcal mol^–1^. But each gives approximately the right answer for the wrong reason. It is interesting that, for this reverse reaction, the reference  exhibits strong curvature in the low-λ region, which is not captured by any of the DFAs. In fact, the DFAs have a qualitatively different shape, suggesting that the λ-dependence of the correlation contributions according to eq 25 could be significantly improved in the reverse dissociation reaction.

**Figure 5:**
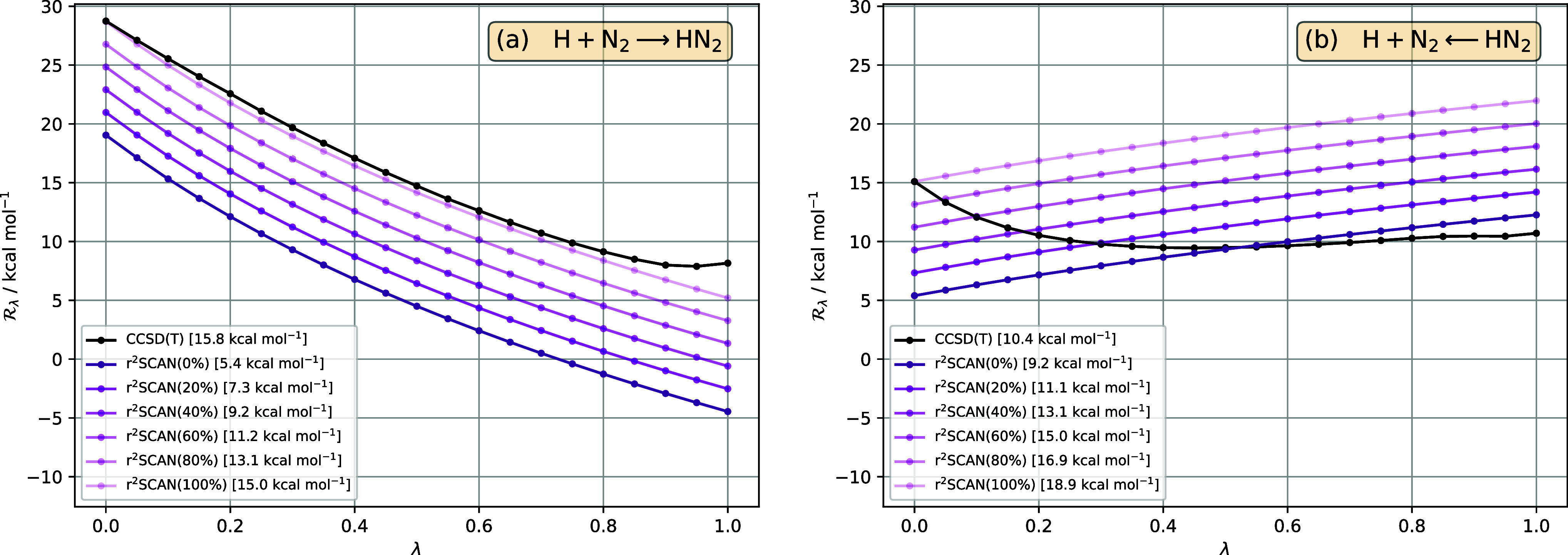
in eq 33, for the reaction H + N_2_ ⇄ HN_2_, as a function of interaction strength λ, in (a) forward and (b) reverse directions, for reference CCSD(T) (black) and r^2^SCAN hybrid functionals, with varying amounts of exact (orbital) exchange indicated in parentheses (purple lines, lighter shading indicates more exact exchange). The pure r^2^SCAN functional is written as r^2^SCAN(0%). All DFA  are calculated for CCSD(T) densities. The barrier for each method—given in the legend—is the area between the  curve and the horizontal axis .

Figure 5a,b present the effect of exact exchange for hybrid versions of r^2^SCAN, for the forward and reverse reactions, respectively. For the forward reaction, the barrier improves steadily as the amount of exchange increases, with an optimal value at 100%, giving an error of just 0.8 kcal mol^–1^. By contrast, for the reverse reaction, an amount between 0 and 20% is optimal; using 100% dramatically overestimates the barrier.

Once again, this difference highlights the key interplay between exchange and correlation. If the correlation functional is accurate (in the sense that the correlation part of eq 25 resembles that of the reference CCSD(T) curve), then the shape of  will be similar to that of the reference curve and so ∼100% exchange will be optimal for the barrier, since only then can the areas from the DFA and reference curves be approximately the same. However, if the correlation functional is poor, then the shape of  will be very different to that of the reference curve and so the optimal amount of exchange must differ from 100%; an accurate barrier will then be a consequence of error cancellation between different λ regions. In terms of the functional components, the residual exchange in the latter case compensates for the error in the correlation functional.

#### 4.1.3 HCN ⇄ CNH and H_2_ + OH ⇄ H_2_O + H

Results for the rearrangement of HCN to CNH and the hydrogen abstraction reaction of H_2_ with OH to form H_2_O and H, together with their reverse reactions, are presented in the Supporting Information. As in Section 4.1.2, the key result is that the shape of  is notably better reproduced by r^2^SCAN in one direction than the other, with the consequence that the optimal amount of exchange is much higher in one direction than the other.

#### 4.1.4 H_2_ + CH_3_ ⇄ H + CH_4_

**Figure 6:**
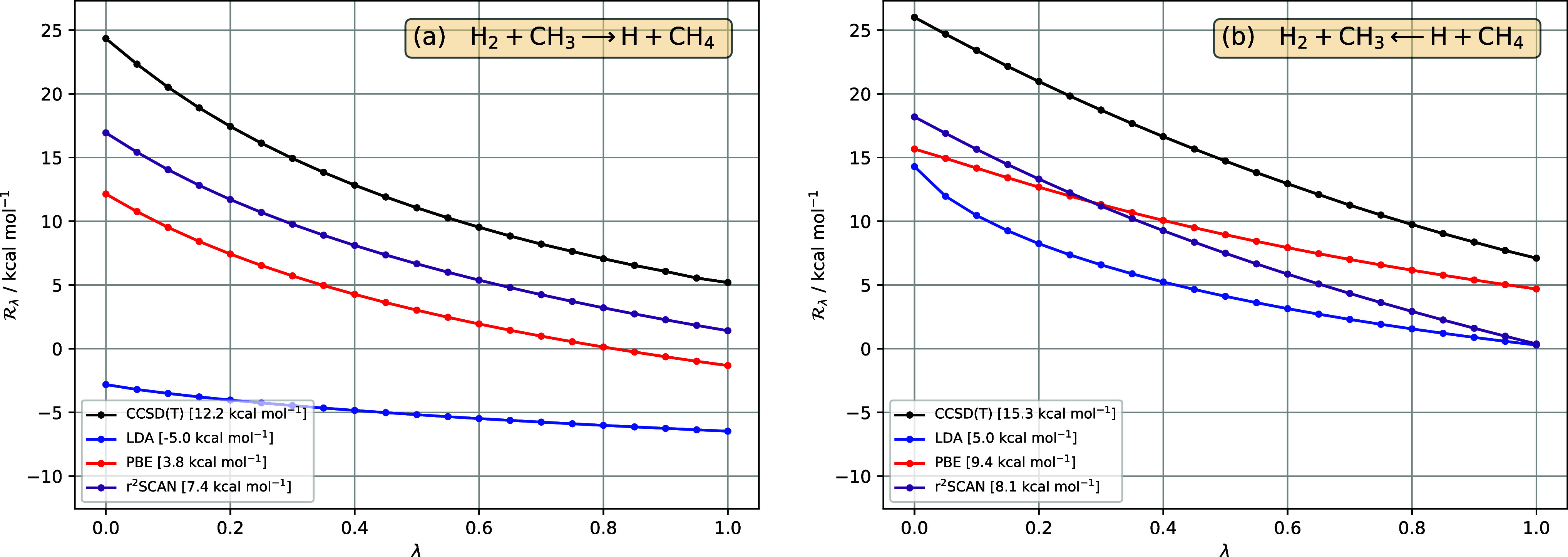
in eq 33, for the reaction H_2_+CH_3_ ⇄ H+CH_4_, as a function of interaction strength λ, in (a) forward and (b) reverse directions, for reference CCSD(T) (black), LDA (blue), PBE (red), r^2^SCAN (purple). All DFA  are calculated for CCSD(T) densities. The barrier for each method—given in the legend—is the area between the  curve and the horizontal axis .

**Figure 7:**
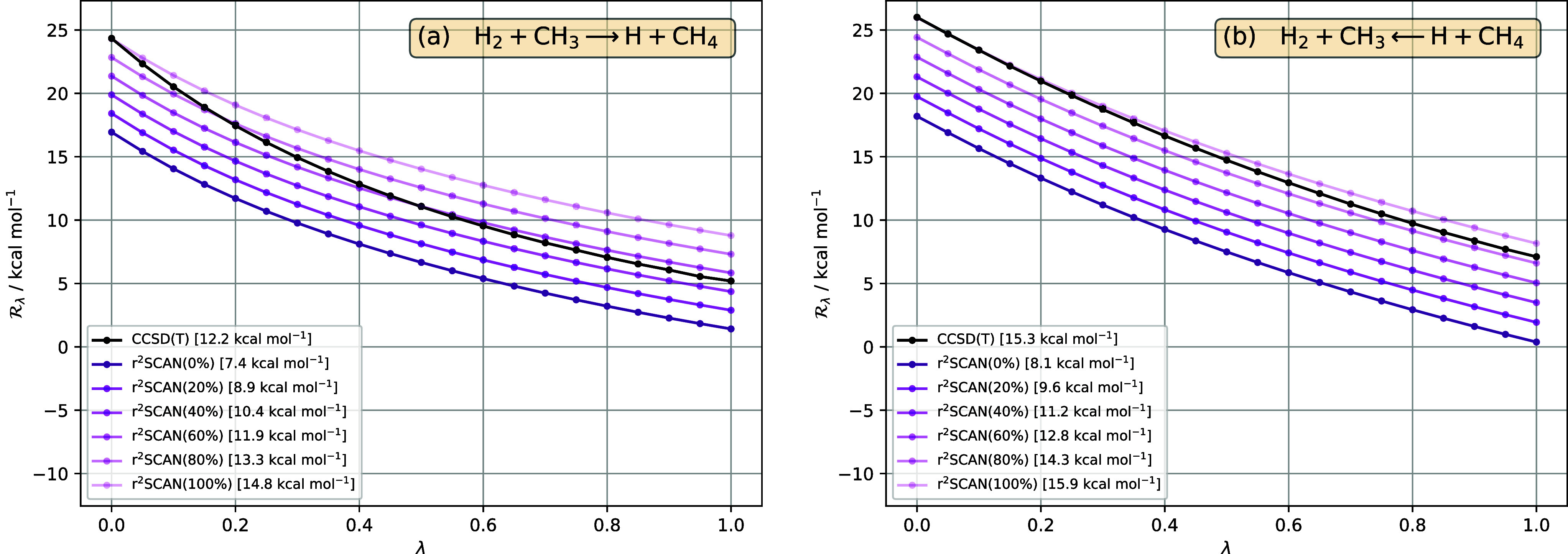
in eq 33, for the reaction H_2_+CH_3_ ⇄ H+CH_4_, as a function of interaction strength λ, in (a) forward and (b) reverse directions, for reference CCSD(T) (black) and r^2^SCAN hybrid functionals, with varying amounts of exact (orbital) exchange indicated in parentheses (purple lines, lighter shading indicates more exact exchange). The pure r^2^SCAN functional is written as r^2^SCAN(0%). All DFA  are calculated for CCSD(T) densities. The barrier for each method—given in the legend—is the area between the  curve and the horizontal axis .

As a final representative reaction from the BH76 set, we consider the hydrogen abstraction reaction of H_2_ with CH_3_ to form H and CH_4_, and the reverse reaction; see Figures 6 and 7. For the forward reaction in Figure 6a, there is marked improvement in the  curve and the barrier from LDA to PBE to r^2^SCAN. The LDA functional exhibits too little curvature and a negative barrier. The  curves for PBE and r^2^SCAN now exhibit a similar shape and so the improvement in  from PBE to r^2^SCAN leads to an improved barrier. For the reverse reaction in Figure 6b, the situation resembles that in Section 4.1.1 and the forward reaction in Section 4.1.2.

Of all the nonsymmetric reactions considered, this is the one where the quality of the shape of  from r^2^SCAN is most similar between the forward and reverse reactions. It follows that both directions benefit from reasonably large amounts of exact exchange, as illustrated in Figure 7. The optimal amount for the forward reaction, just above 60%, is less than for the reverse reaction, which is 80–100%, reflecting the fact that the r^2^SCAN DFA curves are slightly more parallel to the reference CCSD(T) curve for the reverse reaction than for the forward reaction.

### Density-Driven Errors in  and Barriers

4.2

Kaplan et al.,^76^ Kanungo et al.,^77^ and Hernandez et al.^78^ recently investigated the role of functional-driven and density-driven errors in barrier calculations. Their key observation was that the density-driven errors in eq 31 are generally much smaller than the functional-driven errors in eq 32, meaning the overall error in eq 30 is generally dominated by the functional-driven error. However, when they replaced the self-consistent DFA density in these equations with the Hartree–Fock density, the analogue of the density-driven error in eq 31 could become large and positive, leading to some degree of error cancellation with the (unchanged) functional-driven error, meaning the overall error in the barrier could become much smaller.

Here, we investigate this observation from the perspective of  by comparing r^2^SCAN  curves for self-consistent densities, Hartree–Fock densities, and CCSD(T) densities.

#### 4.2.1 H + N_2_ ⇄ HN_2_

**Figure 8:**
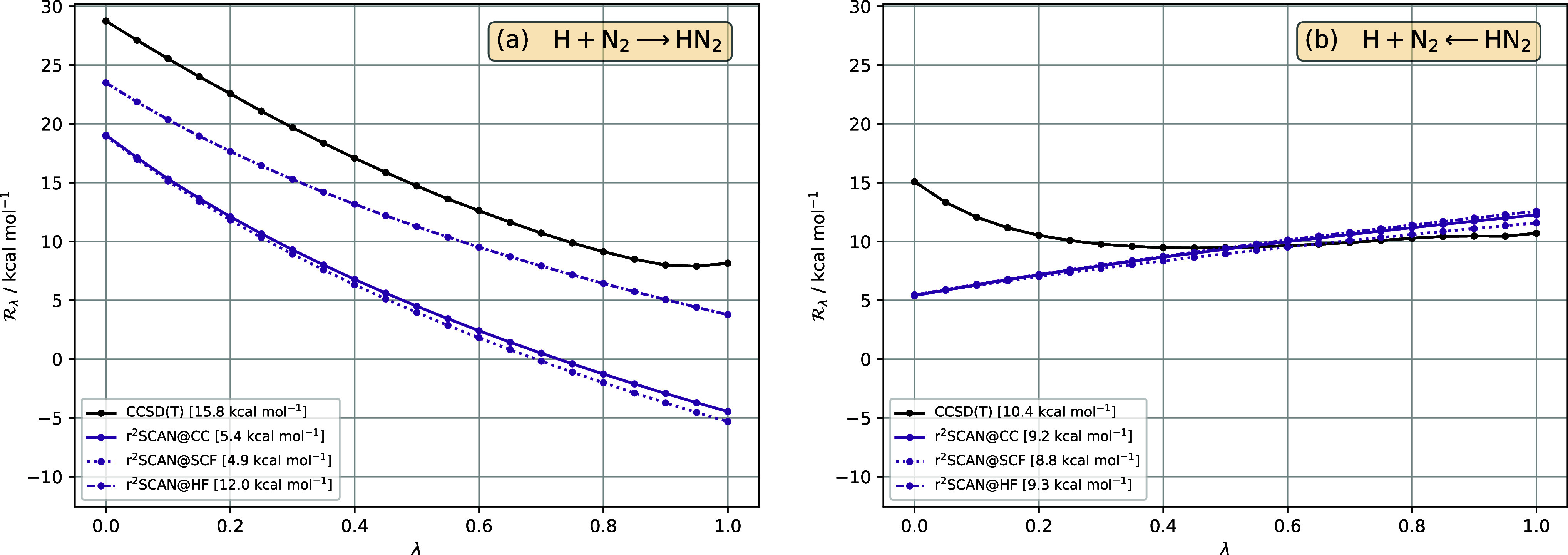
in eq 33, for the reaction H+N_2_ ⇄ HN_2_, as a function of interaction strength λ, in (a) forward and (b) reverse directions, for reference CCSD(T) (black), r^2^SCAN@SCF (purple, dotted), r^2^SCAN@HF (purple, dot-dashed), r^2^SCAN@CC (purple). The barrier for each method—given in the legend—is the area between the  curve and the horizontal axis .

Figure 8 presents the reaction adiabatic-connection integrand  of the forward and reverse H + N_2_ ⇄ HN_2_ reactions for the r^2^SCAN functional, evaluated for three different densities: the self-consistent density (r^2^SCAN@SCF), the Hartree–Fock density (r^2^SCAN@HF), and the CCSD(T) density (r^2^SCAN@CC). We have also included the reference CCSD(T) curve. The CCSD(T) and r^2^SCAN@CC curves have previously been presented in Figure 4, in our discussion of functional-driven errors; in that plot, the r^2^SCAN@CC curve was denoted r^2^SCAN.

For the forward reaction in Figure 8a, the r^2^SCAN@SCF curve is close to the r^2^SCAN@CC curve, indicating that the density-driven error in  is small and that, on average, the r^2^SCAN densities/orbitals are reasonably close to those from CCSD(T). By contrast, the r^2^SCAN@HF curve is notably above the r^2^SCAN@CC curve and closer to the reference CCSD(T) curve, indicating that the Hartree–Fock analogue of the density-driven error is large and positive and goes some way toward canceling the functional-driven error. It is important to note, however, that this improvement is obtained at the expense of a degraded density. For the reverse reaction in Figure 8b, the effect is much less pronounced, with the r^2^SCAN@CC, r^2^SCAN@SCF, and r^2^SCAN@HF curves close together, indicating that the functional-driven error dominates, irrespective of the density used.

The shapes of the three r^2^SCAN curves for the forward reaction in Figure 8a are rather similar, indicating that the correlation scaling property in eq 25 is relatively insensitive to variations in the density. The same is observed for the reverse reaction in Figure 8b. The reason why the Hartree–Fock density has such a pronounced effect for the forward reaction, but not the reverse reaction, is therefore largely because the value of  changes significantly from r^2^SCAN@SCF to r^2^SCAN@HF in the former, but not the latter. For these calculations, differences in  between r^2^SCAN@SCF and r^2^SCAN@HF arise not only from the use of different densities in the exchange terms in  in eq 29, but also from different values of *C*_R_ in eq 28 and *E*_J_ terms in . This contrasts the functional-driven analysis in Section 4.1, where the use of the CCSD(T) density throughout led to the same *C*_R_ and *E*_J_ terms.

An analysis of how the aforementioned terms change from r^2^SCAN@SCF to r^2^SCAN@HF, for both the forward and reverse reaction, offers little insight into the different behavior of . All terms make non-negligible contributions to the change in , the largest magnitudes being the *T*_s_ and *E*_ext_ components of *C*_R_ for the forward and reverse reactions, respectively.

#### 4.2.2 Other Reactions

Results for the hydrogen exchange reaction H + H_2_ → H_2_ + H, for the rearrangement HCN ⇄ CNH, and for the hydrogen abstraction reactions H_2_ + OH ⇄ H_2_O + H and H_2_ + CH_3_ ⇄ H + CH_4_ are presented in the Supporting Information and they show a similar picture: In all cases, the density-driven error in  is small. When the self-consistent density is replaced with the Hartree–Fock density, the effect can be pronounced, leading to some degree of cancellation with the functional-driven error. An analysis of the components of  again offers little insight into the different behaviors.

The  observations in Sections 4.2.1 and 4.2.2 translate into barrier observations, through eq 34, that are fully consistent with those of refs (76−78) while providing additional insight into those observations.

#### 4.2.3 Density-Corrected DFT

The idea of using Hartree–Fock quantities in DFAs to improve barriers is not new. It was proposed in the so-called HF-DFT^79−81^ and a similar idea has recently gained popularity as density-corrected DFT (DC-DFT).^39,82−87^ The application of DC-DFT is somewhat more nuanced, in that the density-sensitivity of a molecule,^84^
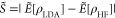
35is quantified by evaluating the absolute change in the total energy for the chosen DFA, when using the LDA density ρ_LDA_ and the Hartree–Fock density ρ_HF_. Since LDA tends to significantly overdelocalize densities, while Hartree–Fock tends to significantly overlocalize densities, this difference gives an indication of the sensitivity of the selected DFA to changes in the density. It has been suggested^84^ that, when *S̃* > 2 kcal mol^–1^ for small molecules, then the system is density-sensitive and DC-DFT should be applied. For the H + N_2_ ⇄ HN_2_ reaction using the r^2^SCAN functional, this threshold is surpassed for N_2_, HN_2_ and the [HN_2_]^‡^ transition state, with values of 2.5, 8.4, and 8.7 kcal mol^–1^, respectively. For the H atom, the density sensitivity value is just 0.3 kcal mol^–1^.

The r^2^SCAN@HF curves in Figure 8 correspond to using the DC-DFT approach for all species, including the H atom. The associated barriers are larger (and more accurate) than the conventional barriers, associated with the r^2^SCAN@SCF curves. The fact that this is so much more pronounced for the forward reaction is fully consistent with the thresholds: while the [HN_2_]^‡^ transition state is density-sensitive with *S̃* = 8.7 kcal mol^–1^, the reactants are relatively density-insensitive, with *S̃* = 0.3 kcal mol^–1^ for H and *S̃* = 2.5 kcal mol^–1^ for N_2_. As a result, the use of the HF density raises the energy of the transition state relative to the (variationally optimal) self-consistent energy by more than it raises the energy of the reactants, resulting in a significant increase in the r^2^SCAN@HF barrier compared with that of r^2^SCAN@SCF. For the reverse reaction, the transition state is only marginally more density-sensitive than the reactant, with *S̃* values of 8.7 and 8.4 kcal mol^–1^ respectively. As a result the effect of using the HF density is to raise the energy of the reactant and transition state by approximately the same amount, leading to relatively little change in the barrier.

In a recent study, Hernandez et al.^78^ also examined the issue of error cancellations when applying DC-DFT to calculate barriers, reaching similar conclusions. As part of their study, the authors also proposed a second criterion to check that the Hartree–Fock density is reasonable for use in DC-DFT. Specifically, the degree of spin-contamination |⟨*S*^2^⟩_HF_ – ⟨*S*^2^⟩_exact_| should not exceed 10% for open-shell systems. For the [HN_2_]^‡^ transition state, this value is 20.1%, while for the product HN_2_, it is 17.1%, suggesting that DC-DFT may not be suitable for use in either the forward or reverse reaction. In the present work, calculations at the r^2^SCAN@HF level are used merely to quantify the effect of changing the density, which is of interest regardless of whether DC-DFT should be applied practically. Finally, it should also be noted that the criteria for applying DC-DFT are heavily sensitive to the choice of DFA. For the 15 systems comprising the 5 reactions studied in this work, the combined *S̃* and ⟨*S*^2^⟩ criteria suggest that only 5 systems should be corrected for r^2^SCAN. This increases to 11 for both the LDA and PBE functionals.

## 5 Conclusions

The calculation of classical reaction barriers has long been challenging for semilocal DFAs, which have a tendency to severely underestimate these quantities. We have examined these shortcomings from a new perspective—namely, that of the density-fixed adiabatic connection, which links the Kohn–Sham noninteracting system to the physical interacting system. A reaction adiabatic-connection integrand, , was introduced, such that integration over the interaction strength, λ, from 0 to 1 yields the barrier, meaning the barrier can be easily visualized as the area under a plot of  vs λ.

Initially, we focused on functional-driven errors by comparing reference  curves obtained from CCSD(T) Lieb maximizations with density-functional approximations to these curves for LDA, PBE, and r^2^SCAN, determined using coordinate scaling for CCSD(T) densities. By fixing the densities to be those obtained from the CCSD(T) wave function, our analysis provides a simple way to visualize and understand functional-driven errors and trends in barriers from approximate functionals, while allowing a clean separation of the role of exchange and correlation contributions to the barrier. Specifically, the accuracy of  is determined entirely by the accuracy of the exchange DFA, while the shape of  is determined entirely by the correlation functional.

As may be expected, the value of  tended to improve from LDA to PBE to r^2^SCAN. Increasing the fraction of exact (orbital) exchange to form hybrid functionals has a particularly simple effect on the  curves: it shifts them vertically, improving the value of , until the reference CCSD(T) value is recovered with 100% exchange.

Obtaining the correct shape of  was more challenging. In some reactions, the shape of  was reasonably well described, in which case increasing the amount of exact exchange to close to 100% led to a significant improvement in the barrier. However, in other cases the shape of  was poorly described and, while a modest increase in the amount of exact exchange did lead to a nominal improvement in the barrier due to cancellation of errors in high and low λ regions, this is nothing more than error compensation between the exchange and correlation contributions. We note that for the four reactions where the forward and reverse directions are different, the shape of  is best described for the direction with the larger barrier, i.e. the direction with the later transition state. However, the amount of data is limited and so further investigation is required to establish if this trend is more widely applicable.

These results illustrate how simply introducing larger amounts of exact exchange may not be a reliable approach to generate improved functionals for barriers, especially since the amount required for forward and reverse directions of the same reaction may be significantly different. To make significant progress, the shape of  must be captured more accurately. Since the shape is determined *entirely* by the correlation functional and its scaling properties according to eq 25, it would be fruitful to consider design of functionals parametrized to more accurately reproduce the shape of . The results reported here present a first step toward benchmark numerical data that may be useful for this purpose and this will be pursued in future work.

We then considered density-driven errors, comparing self-consistent r^2^SCAN results for  with those evaluated using r^2^SCAN for the reference CCSD(T) density. The self-consistent  curves remained close to those evaluated for the reference CCSD(T) density, indicating that the density-driven error is small and that, at least on average, the r^2^SCAN densities/orbitals of each species involved in the reactions studied are close to the CCSD(T) ones. Using the Hartree–Fock density instead, as is done in HF-DFT and DC-DFT, could lift the , bringing it closer to the reference CCSD(T) curve, reflecting some degree of cancellation with the functional-driven errors, consistent with recent studies.^76−78^ However, given that the associated improvements in the barriers are not a result of an improved density, barriers obtained using HF-DFT and DC-DFT should be treated with some caution.

Finally, we also considered the use of the density-sensitivity measure *S̃* of eq 35 and the spin-contamination criterion of Hernandez et al.^78^ Our  and barrier results for a representative reaction are fully consistent with the values of *S̃*, whereby similar sensitivities in the reactant/transition state lead to a small effect on the reverse barrier, whereas very different sensitivities in reactant/transition state led to a much larger effect on the forward barrier.

In this work, we have focused on simple semilocal approximations, since these represent some of the most cost-effective methods in widespread application. However, range-separated hybrids often give rise to much improved barriers when tested on, for example, the BH76 benchmark set. An interesting avenue for future work is therefore to use a generalized adiabatic connection to study such functional forms. Indeed, such calculations can be carried out with the framework used in the present work, as shown in ref (88). Alternative adiabatic connections, where the electron density is not held fixed, could also be considered in a similar manner, for example the potential-fixed adiabatic connection in ref (43)., or the Mo̷ller–Plesset adiabatic connection in ref (89).; the latter may give additional insight into the performance of approaches that use a Hartree–Fock reference.

We note that errors in barriers are often attributed to self-interaction, which functionals with increased orbital-dependent exchange and approaches such as HF-DFT/DC-DFT are thought to help mitigate. The use of explicit self-interaction corrections has been explored extensively in the recent literature and it would be interesting to analyze how  is influenced by these approaches.

Finally, our approach for defining an adiabatic-connection integrand for reaction barriers could be applied to many other properties, including the majority of those in the GMTKN55 database. Future work will pursue the construction of adiabatic-connection data sets for a range of quantities, providing extensive data to benchmark new density-functional approximations.

## Data Availability

The data that supports the findings of this work are available within the article.

## Appendix A

### GDIIS Optimization of *G*_λ,ρ_[**b**]

The GDIIS algorithm^50^ is defined by choosing an approximation to the error vectors **e**_*i*_ that define the difference between the potential coefficients **b**_*i*_ at a particular iteration *i* and the converged potential coefficients **b***, such that **b**_*i*_ = **b*** + **e**_*i*_. The error vectors can be approximated by those for a quadratic function,

A1

where **H** and **g** are the Hessian and gradient with respect to the coefficients **b**, respectively. A set of potential expansion coefficients and error vectors are generated for *m* iterations, defining an *m*-dimensional iterative subspace. Minimisation of the residuum vector ∑_*i*_*c*_*i*_**e**_*i*_ subject to the constraint ∑_*i*_*c*_*i*_ = 1 leads to the DIIS equations,

A2

where *B*_*ij*_ = ⟨**e**_*i*_|**e**_*j*_⟩ is the scalar product of the error vectors **e**_*i*_ and **e**_*j*_ and ζ is a Lagrange multiplier. The potential coefficients at the next iteration can then be calculated according to

A3

where the coefficients *c*_*i*_ are determined from A2 and define an interpolated set of coefficients (the first term of A3) and an interpolated gradient (in parentheses in the second term of A3). The potential coefficients for the next step **b**_*m*+1_ then correspond to the interpolated coefficients updated by a Newton step constructed using an approximation to the Hessian **H** and the interpolated gradient. In the present work, we use the noninteracting Hessian, given by eq 21 at λ = 0.^49^ Since the quadratic approximation of A1 is only valid close to the maximum of *G*_λ,ρ_[**b**], the GDIIS algorithm is switched on when the Euclidean norm of **g**_*i*_ falls below 10^–4^ a.u.

## Supporting Information Available

Details of Lieb maximizations, convergence of reaction barriers with respect to basis set and excitation level in coupled-cluster calculations, and functional-driven and density-driven errors in  and barriers for additional reactions (PDF)
