# Supplementary material for: Classical Reaction Barriers in DFT: An Adiabatic-Connection Perspective
Source: J Chem Theory Comput. 2024 Dec 23;21(1):124–37. doi: 10.1021/acs.jctc.4c01038 (PMC11736800; doi:10.1021/acs.jctc.4c01038)
Supplement: Supplementary file 1 — ct4c01038_si_001.pdf [file ct4c01038_si_001.pdf]

Supporting Information

Classical reaction barriers in DFT: An adiabatic-connection perspective

Andrew M. Wibowo-Teale*[,1], Bang C. Huynh[1], Trygve Helgaker*[,2], and David J. Tozer*[,3]

[1]School of Chemistry, University of Nottingham, University Park, Nottingham NG7 2RD, UK
[2]Hylleraas Centre for Quantum Molecular Sciences, Department of Chemistry, University of Oslo, P. O. Box 1033 Blindern, N-0315 Oslo, Norway
[3]Department of Chemistry, Durham University, South Road, Durham DH1 3LE, UK

November 27, 2024

1 Lieb Maximisations

To confirm that the Lieb maximisations yield densities of sufficient accuracy for all λ, we evaluate the difference between the classical Coulomb energy evaluated on the converged density and the target coupled-cluster density $|E_J[\rho_\lambda] - E_J[\rho_{CC}]|$. For the relatively modest cc-pCVTZ basis set used in the present work these values do not exceed 10^{-4} hartree (approximately 0.06 kcal mol^{-1}). As a further check, the total energy is evaluated from the Kohn-Sham energy components available at $\lambda = 0$: T_s, E_{nn}, E_{ne}, E_J and the coupling constant integrated exchange correlation energy $E_{xc} = \int_0^1 \mathcal{W}_{xc,\lambda} d\lambda$. The energies evaluated in this manner show discrepancies with the standard coupled-cluster energies on the order of 10^{-4} hartree for all systems in the present work. These discrepancies can be further reduced by increasing the cardinal number of basis set, however, we choose the cc-pCVTZ basis as a good compromise between sufficient accuracy and computational cost.

2 Reaction Barriers

In the main manuscript, we present reaction barriers at the CCSD(T)/cc-pCVTZ level of theory that are in agreement with reference data to within 1.5 kcal mol^{-1}, and in the vast majority of cases within better than 1.0 kcal mol^{-1}. Detailed convergence of the reaction barriers considered with respect to basis set size and excitation level in coupled-cluster calculations is presented in the table below.

Table S1: The reaction barriers considered in the present study calculated at the CCSD and CCSD(T) levels of theory with the cc-pCVNZ basis sets, for cardinal numbers $N = 3 - 5$. Complete basis set (CBS) extrapolated values and W2-F12 values from the GMTKN55 database are included for comparison. All values in kcal mol^{-1}.

Reaction	Barrier	CCSD $N=3$	CCSD $N=4$	CCSD $N=5$	CCSD(T) $N=3$	CCSD(T) $N=4$	CCSD(T) $N=5$	Reference CBS[a]	Reference W2-F12[b]
$H + H_2 \longrightarrow H_2 + H$	Fwd.	10.3	10.1	10.1	10.0	9.8	9.7	9.7	9.7
$H + N_2 \rightleftharpoons HN_2$	Fwd.	16.0	15.5	15.4	15.6	15.0	14.8	14.7	14.6
	Rev.	11.1	11.6	11.7	10.2	10.6	10.7	10.8	10.9
$HCN \rightleftharpoons CNH$	Fwd.	48.4	48.5	48.5	48.0	48.1	48.1	48.1	48.1
	Rev.	33.8	33.9	33.9	33.0	33.0	33.0	33.0	33.0
$H_2 + OH \rightleftharpoons H_2O + H$	Fwd.	8.2	7.6	7.4	6.6	5.8	5.5	5.1	5.2
	Rev.	20.6	21.3	21.6	20.6	21.3	21.5	21.6	21.6
$H_2 + CH_3 \rightleftharpoons H + CH_4$	Fwd.	13.5	13.4	13.4	12.2	12.0	12.0	11.9	11.9
	Rev.	15.6	15.5	15.4	15.3	15.1	15.0	15.0	15.0

[a] The complete basis set (CBS) limit estimates are obtained using the cc-pCV5Z Hartree-Fock energy (following the recommendations of Ref. [1]), and the two-point extrapolation formula for the correlation energy in Ref. [2] using the cc-pCVQZ and cc-pCV5Z CCSD(T) correlation energies.
[b] The W2-F12 values are taken from the BH76 set of the GMTKN55 database [3, 4, 5, 6], where they were determined using the protocol of Ref. [7].

A number of general observations can be made from these data. Firstly, coupled-cluster calculations with cardinal number $N = 3$ in the cc-pCVNZ series of basis sets are reasonably well converged. The $N = 3$ CCSD and CCSD(T) barriers agree with the corresponding $N = 5$ values to within 1.1 kcal mol^{-1}. However, comparing with reference data it is clear that the consideration of triple excitations is necessary to achieve high accuracy for reaction barriers and the values systematically improve when moving from CCSD to CCSD(T) for all N. As reference data, we include an estimate of the complete basis set (CBS) limit obtained using the cc-pV5Z Hartree-Fock energy (following the recommendations of Ref. [1]), and the two-point extrapolation formula for the correlation energy in Ref. [2] using the cc-pCVQZ and cc-pCV5Z CCSD(T) correlation energies. These values agree remarkably well (to within 0.1 kcal mol^{-1}) with W2-F12 values taken from the GMTKN55 database [3, 4, 5, 6], which were calculated using the protocol of Ref. [7]. This suggests that additional scalar relativistic, spin-orbit and diagonal Born-Oppenheimer corrections incorporated in the W2-F12 protocol play a limited role for the reactions involving systems composed of relatively light elements considered in this work.

Overall, these data suggest that the CCSD(T)/cc-pVTZ level of theory represents a reasonable compromise between accuracy and computational efficiency. Considering the relatively large errors in reaction barriers for semi-local density-functional approximations, the reaction adiabatic connection curves determined at this level of theory are expected to provide a reasonable benchmark.

3 Functional-driven errors in $\mathcal{R}_\lambda$ and barriers

HCN $\rightleftarrows$ CNH

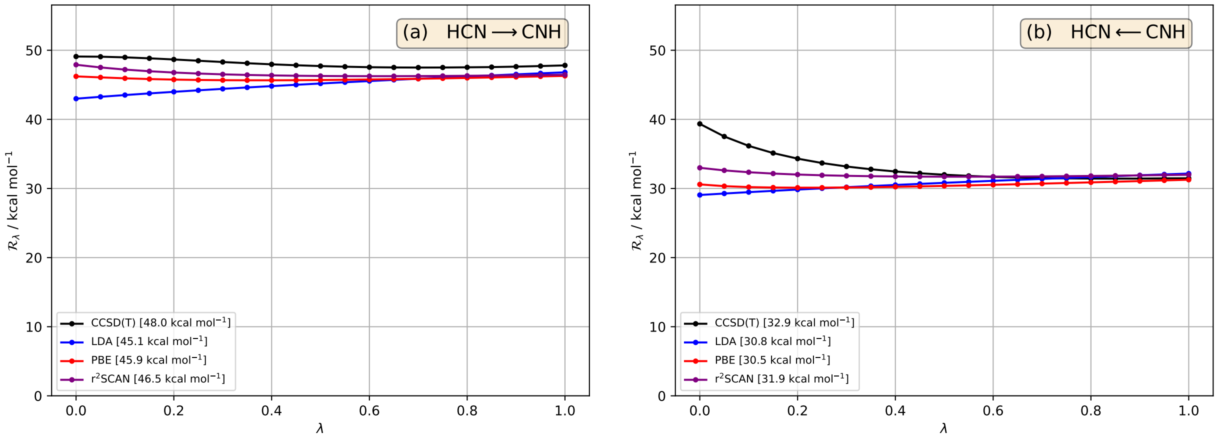

Figure S1: $\mathcal{R}_\lambda$, for the reaction HCN $\rightleftarrows$ CNH, as a function of interaction strength λ, in (a) forward and (b) reverse directions, for reference CCSD(T) (black), LDA (blue), PBE (red), r^{2}SCAN (purple). All DFA $\mathcal{R}_\lambda$ are calculated for CCSD(T) densities. The barrier for each method—given in the legend—is the area between the $\mathcal{R}_\lambda$ curve and the horizontal axis ($\mathcal{R}_\lambda = 0$).

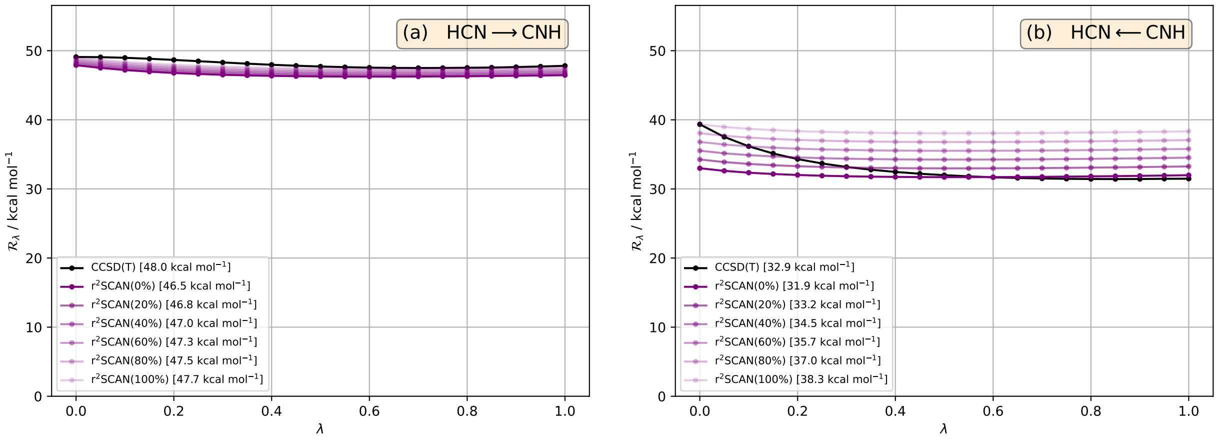

Figure S2: $\mathcal{R}_\lambda$, for the reaction HCN $\rightleftarrows$ CNH, as a function of interaction strength λ, in (a) forward and (b) reverse directions, for reference CCSD(T) (black) and r^{2}SCAN hybrid functionals, with varying amounts of exact (orbital) exchange indicated in parentheses (purple lines, lighter shading indicates more exact exchange). The pure r^{2}SCAN functional is written as r^{2}SCAN(0%). All DFA $\mathcal{R}_\lambda$ are calculated for CCSD(T) densities. The barrier for each method—given in the legend—is the area between the $\mathcal{R}_\lambda$ curve and the horizontal axis ($\mathcal{R}_\lambda = 0$).

$H_2 + OH \rightleftharpoons H_2O + H$

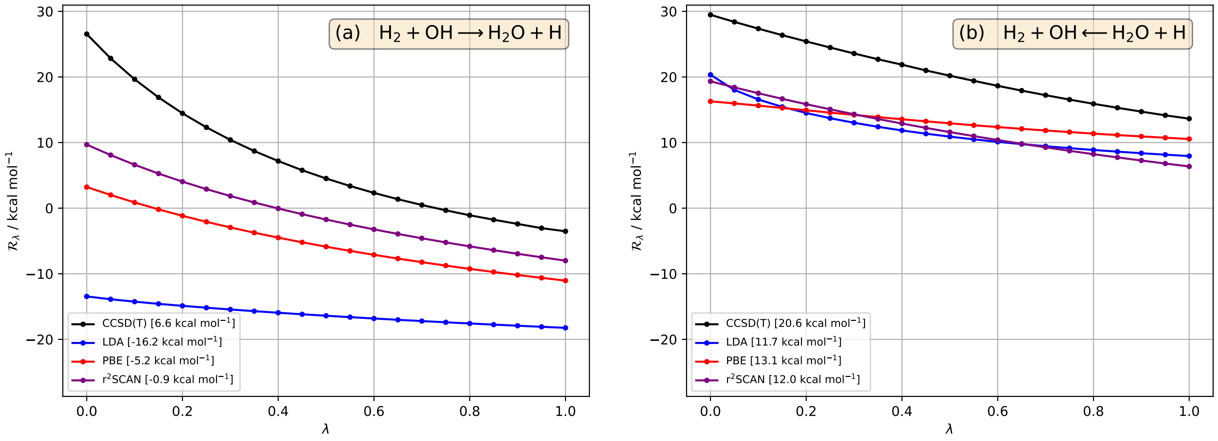

Figure S3: $\mathcal{R}_\lambda$, for the reaction $H_2 + OH \rightleftharpoons H_2O + H$, as a function of interaction strength λ, in (a) forward and (b) reverse directions, for reference CCSD(T) (black), LDA (blue), PBE (red), r^2SCAN (purple). All DFA $\mathcal{R}_\lambda$ are calculated for CCSD(T) densities. The barrier for each method—given in the legend—is the area between the $\mathcal{R}_\lambda$ curve and the horizontal axis ($\mathcal{R}_\lambda = 0$).

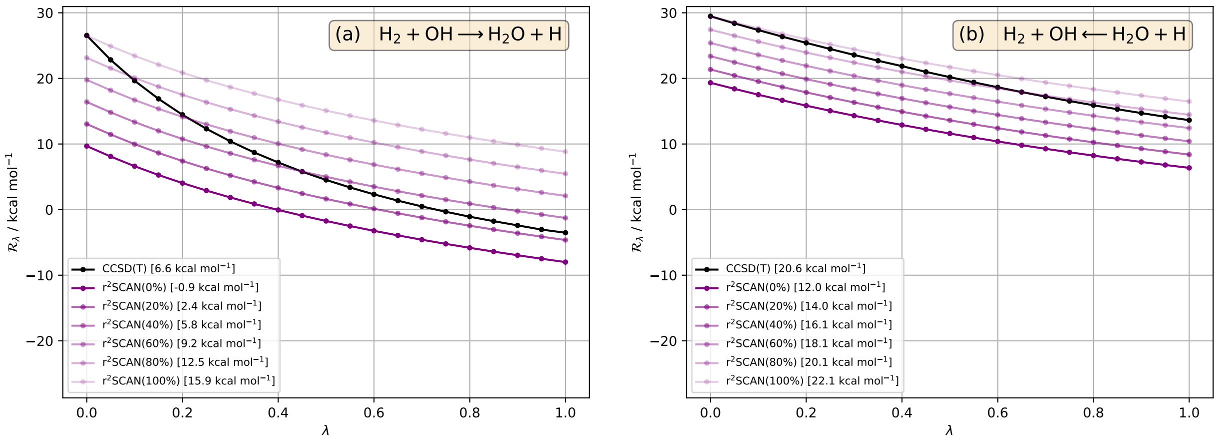

Figure S4: $\mathcal{R}_\lambda$, for the reaction $H_2 + OH \rightleftharpoons H_2O + H$, as a function of interaction strength λ, in (a) forward and (b) reverse directions, for reference CCSD(T) (black) and r^2SCAN hybrid functionals, with varying amounts of exact (orbital) exchange indicated in parentheses (purple lines, lighter shading indicates more exact exchange). The pure r^2SCAN functional is written as r^2SCAN(0%). All DFA $\mathcal{R}_\lambda$ are calculated for CCSD(T) densities. The barrier for each method—given in the legend—is the area between the $\mathcal{R}_\lambda$ curve and the horizontal axis ($\mathcal{R}_\lambda = 0$).

4 Density-driven errors in $\mathcal{R}_\lambda$ and barriers

$\mathbf{H + H_2 \rightleftarrows H_2 + H}$

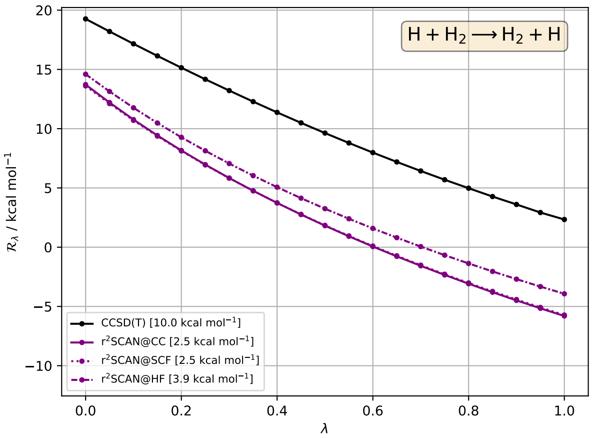

Figure S5: $\mathcal{R}_\lambda$, for the reaction $\mathrm{H + H_2 \rightleftarrows H_2 + H}$, as a function of interaction strength λ, for reference CCSD(T) (black), r^{2}SCAN@SCF (purple, dotted), r^{2}SCAN@HF (purple, dot-dashed), r^{2}SCAN@CC (purple). The barrier for each method—given in the legend—is the area between the $\mathcal{R}_\lambda$ curve and the horizontal axis ($\mathcal{R}_\lambda = 0$).

$\mathbf{HCN \rightleftarrows CNH}$

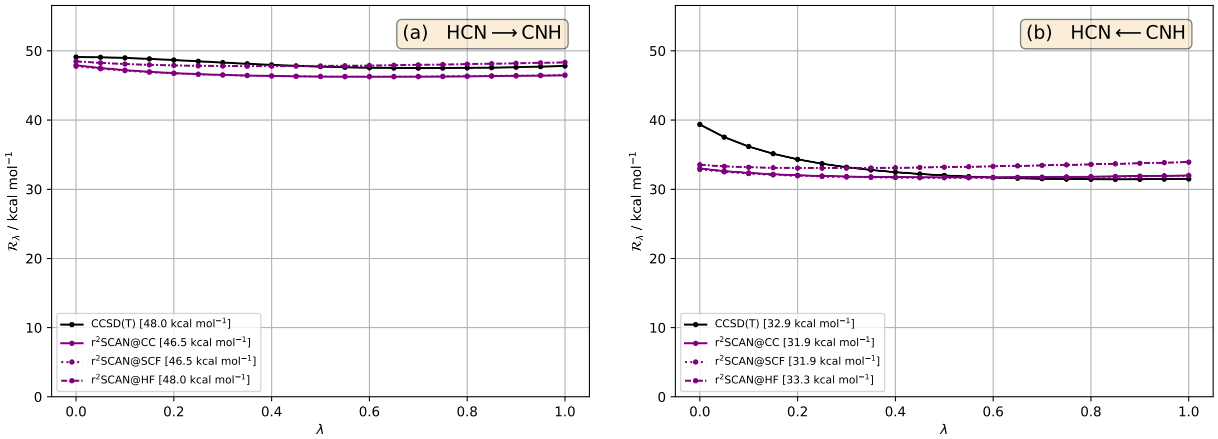

Figure S6: $\mathcal{R}_\lambda$, for the reaction $\mathrm{HCN \rightleftarrows CNH}$, as a function of interaction strength λ, in (a) forward and (b) reverse directions, for reference CCSD(T) (black), r^{2}SCAN@SCF (purple, dotted), r^{2}SCAN@HF (purple, dot-dashed), r^{2}SCAN@CC (purple). The barrier for each method—given in the legend—is the area between the $\mathcal{R}_\lambda$ curve and the horizontal axis ($\mathcal{R}_\lambda = 0$).

$H_2 + OH \rightleftarrows H_2O + H$

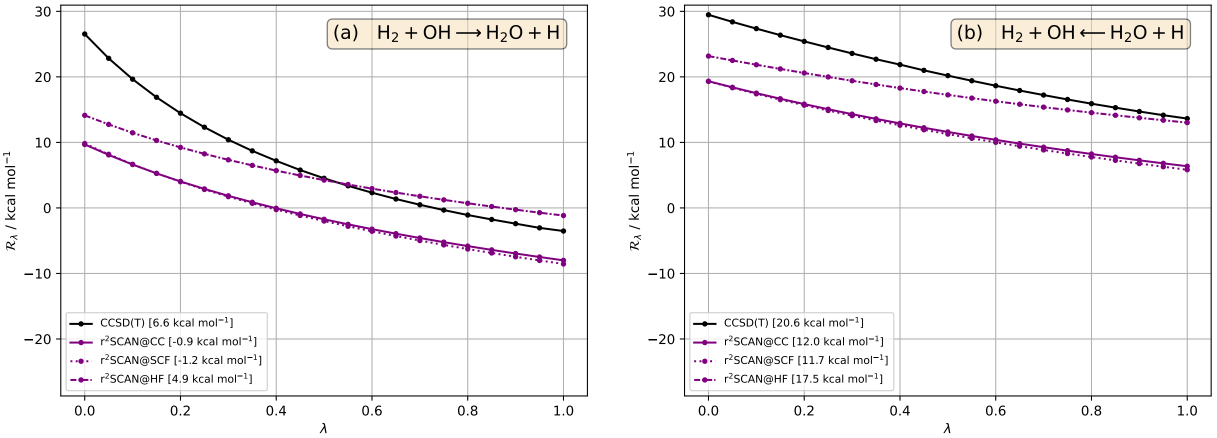

Figure S7: $\mathcal{R}_\lambda$, for the reaction $H_2 + OH \rightleftarrows H_2O + H$, as a function of interaction strength λ, in (a) forward and (b) reverse directions, for reference CCSD(T) (black), r^{2}SCAN@SCF (purple, dotted), r^{2}SCAN@HF (purple, dot-dashed), r^{2}SCAN@CC (purple). The barrier for each method—given in the legend—is the area between the $\mathcal{R}_\lambda$ curve and the horizontal axis ($\mathcal{R}_\lambda = 0$).

$H_2 + CH_3 \rightleftarrows H + CH_4$

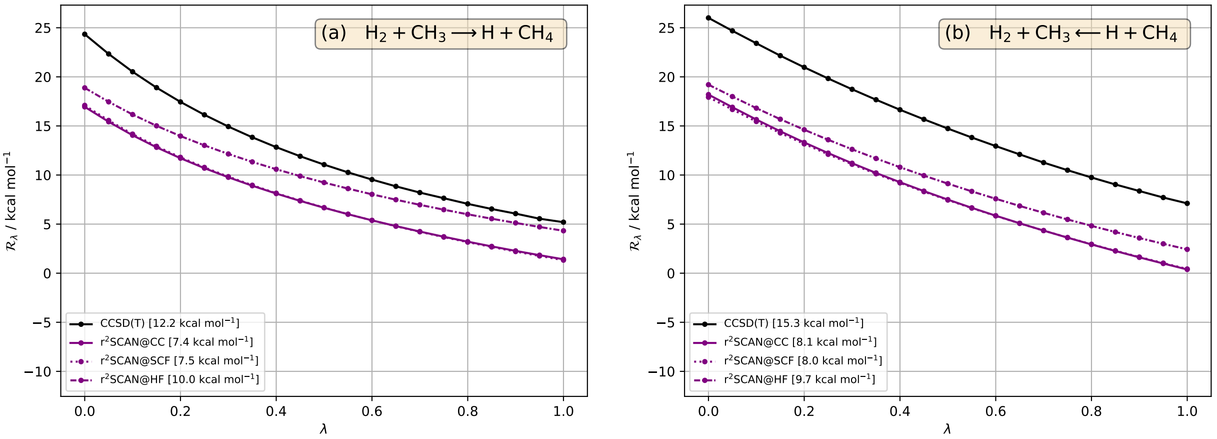

Figure S8: $\mathcal{R}_\lambda$, for the reaction $H_2 + CH_3 \rightleftarrows H + CH_4$, as a function of interaction strength λ, in (a) forward and (b) reverse directions, for reference CCSD(T) (black), r^{2}SCAN@SCF (purple, dotted), r^{2}SCAN@HF (purple, dot-dashed), r^{2}SCAN@CC (purple). The barrier for each method—given in the legend—is the area between the $\mathcal{R}_\lambda$ curve and the horizontal axis ($\mathcal{R}_\lambda = 0$).

References

[1] A. Halkier, T. Helgaker, P. Jørgensen, W. Klopper, and J. Olsen, *Chem. Phys. Lett.* **302**, 437–446 (1999).

[2] A. Halkier, T. Helgaker, P. Jørgensen, W. Klopper, H. Koch, J. Olsen, and A. K. Wilson, *Chem. Phys. Lett.* **286**, 243–252 (1998).

[3] Y. Zhao, B. J. Lynch, and D. G. Truhlar, *Phys. Chem. Chem. Phys.* **7**, 43 (2005).

[4] Y. Zhao, N. González-García, and D. G. Truhlar, *J. Phys. Chem. A* **109**, 2012–2018 (2005).

[5] L. Goerigk and S. Grimme, *J. Chem. Theory Comput.* **6**, 107–126 (2009).

[6] L. Goerigk, A. Hansen, C. Bauer, S. Ehrlich, A. Najibi, and S. Grimme, *Phys. Chem. Chem. Phys.* **19**, 32184–32215 (2017).

[7] A. Karton and J. M. L. Martin, *J. Chem. Phys.* **136**, 124114 (2012).
